# Book Notices

**Published:** 1899-01

**Authors:** 


### BOOK NOTICES.

Essentials of Homœopathic Therapeutics; being a quiz compend of the application of homœopathic remedies to diseased states. A companion to the *Essentials of Homœopathic Materia Medica.* Arranged and compiled especially for the use of students of medicine by W. A. Dewey, M. D. Second edition, revised and enlarged. Philadelphia: Bœricke & Tafel, 1898. Price, cloth, $1.50 net; by mail, $1.59.

The revelations made by State examining boards of the shortcomings of graduates who apply for licenses to practice; their especial deficiency in the particular branch of homœopathic therapeutics and homœopathic materia medica show the urgent need of a systematic and thorough scheme of instruction in this important element of medical education.

The failures in materia medica, before the medical examining boards, of applicants who hold the diplomas of colleges show how lamentably the colleges fail in their duties in this one particular. Therefore, the attention of college professors should be concentrated on the solution of this one problem.

One attempt at this solution is to be found in the little volume now before us.

It consists of a series of questions and answers, the whole being devoted to the subject of improving the student's knowledge of materia medica.

We have looked over the pages of the book with great interest, and found ourselves urgently in need of the instruction to be found there. An earnest student will at once possess himself of the book, and commit to memory everything it contains, with the satisfactory feeling that he is better equipped for a contest with the examining boards and more likely to come off victorious.

Keynotes and Characteristics, with comparisons, of some of the leading remedies of the Materia Medica. By H. C. Allen, M. D., Professor of Materia Medica in Hering Medical College and Hospital, Chicago. Philadelphia: Bœricke & Tafel. 1898. Price, cloth, $1.25 net; by mail, $1.32.

This little volume contains the old, true, and tried keynote indications ot the homœopathic materia medica. They are about the same as those published in the editorials of The Homœopathic Physician under the name of "Dr. Guernsey's Keynotes."

They are systematically arranged in groups under the names of the remedies to which they belong, and the remedies are arranged in alphabetical order. They should have been followed by an index repertory, which would have vastly increased the value of the book. Like the *Homœopathic Therapeutics*, by Dr. Dewey, reviewed in this number, the contents of Allen's book should be memorized by every student, and the book itself should be placed among the reference books at the hand of every advanced practitioner.

The copy possessed by the editor, will, from this time, be found on the shelves where he keeps his books of consultation in the daily treatment of cases. This is our testimony to the usefulness of this book.

Another item of value of this book is that it adds yet another effort to the scheme we have been advocating for a system of materia medica instruction of graduates, which the reader will find mentioned in the review of Dr. Dewey's book, entitled, *Essentials of Homœopathic Therapeutics*, published in this number.

A Primer of Psychology and Mental Disease. For use in training schools for attendants and nurses, and in medical classes. By C. B. Burr, M. D. Second edition, thoroughly revised. Philadelphia, New York, and Chicago: The F. A. Davis Co., Publishers. 1898.

This clever little book by the medical director of Oak Grove Hospital for nervous and mental diseases, at Flint, Michigan, is intended to help students in classes in the Training School the better to understand the special subjects treated in the lectures on psychology.

Starting with a statement of the definition of psychology and life, it proceeds to analyze, in the simplest possible way, the operations and phenomena of mind, and make them intelligible. It attempts a definition of insanity which is as follows: "A prolonged departure from the individual's normal standard of thinking, feeling, and acting." Without going into a discussion of this definition we may say that it is the best we have seen.

All the statements in the book are concise, and all the subjects are treated in the briefest possible way, though we must add, they are clear. It is a suitable volume to have at hand when attending lectures on psychology, as it really amounts to a syllabus with the addition of the most important explanations so they may be memorized.

It is a good little volume for student and practitioner alike to acquire clear primary principles of the science of psychology.

